# Case Report: Syndrome of Remitting Seronegative Symmetrical Synovitis with Pitting Edema—A Rare but Treatable Condition in Palliative Medicine

**DOI:** 10.1089/pmr.2021.0070

**Published:** 2022-12-26

**Authors:** L. Merkle Moore, Jennifer M. Brouner, Nelly Grigorian, R.J. Leach, Steven J. Baumrucker

**Affiliations:** ^1^ ETSU Quillen College of Medicine, Johnson City, Tennessee, USA.; ^2^ Wellstar Health System Palliative Medicine Fellowship, Marietta, Georgia, USA.; ^3^ Palliative Medicine, Ballad Health System, Kingsport, Tennessee, USA.

**Keywords:** bone pain, corticosteroids, palliative medicine, paraneoplastic, RS3PE, synovitis

## Abstract

The syndrome of remitting seronegative symmetrical synovitis with pitting edema (RS3PE) is a rare diagnosis that is often missed due to lack of both definitive diagnostic criteria and awareness of the disease. This case report describes a patient with chronic lymphocytic leukemia whose diagnosis of RS3PE was possibly delayed due to concomitant treatment-related arthralgias. The pathophysiology, presentation, and treatment of RS3PE are discussed. Greater awareness of malignancy-related RS3PE is crucial from a palliative care perspective as typical opioid pain management will prove ineffective and delay appropriate treatment.

## Introduction

The syndrome of remitting seronegative symmetrical synovitis with pitting edema (RS3PE) is a rare diagnosis that is often missed due to a lack of both definitive diagnostic criteria and awareness of the disease. The medical literature suggests that the syndrome is often associated with neoplastic states.^1^ RS3PE presents as arthritis with pitting edema, synovitis, and is seronegative for rheumatological factor. It responds well to treatment with glucocorticoids.

This report demonstrates a case in which a patient with a malignancy developed joint pain, and because of the rarity of the syndrome, its diagnosis may have been delayed. It is critical that palliative medicine providers be aware of this syndrome, as early diagnosis and treatment will improve pain management in affected patients and reduce burden of ineffective opioid treatment.

## Case Report

A 47-year-old male with a history of chronic back pain, diverticulitis, chronic lymphocytic leukemia (CLL), and an extensive history of diverticulitis, IBS, and colitis suspected to be secondary to CLL and a suspected concomitant neuroendocrine tumor presented with new onset bone pain and was referred to palliative medicine for symptom management. The patient also complained of worsening arthralgia in the hips and knees, unresponsive to escalating opioid doses.

After failure of a trial of oral naproxen, the patient was started on 2 mg hydromorphone every six hours as needed that was titrated over several weeks to 4 mg every four hours as needed without relief. The clinical picture was complicated by daily dosing of ibrutinib, 280 mg orally, a Bruton's tyrosine kinase (BTK) inhibitor, which is associated with arthralgias, for treatment of his CLL.^2^ As a result, his worsening joint pain was initially diagnosed as a treatment-related adverse effect. After dose adjustments of the BTK inhibitor failed to relieve his symptoms, the treatment was ultimately discontinued.

However, two months after ibrutinib cessation, the patient continued to report joint pain and a new and sudden onset of nonerythematous induration of his hands without associated pedal edema. He noted that the induration “waxed and waned at random.” Physical examination revealed pitting edema of the hands and wrists with mild-to-moderate tenderness to palpation. No erythema, mottling, temperature change, or loss of hair was noted. There was no skin thickening, nor desquamation.

At that time, the differential was broadened to include an underlying rheumatological disease, and he was referred to a rheumatologist for further evaluation. An antinuclear antibody (ANA), rheumatoid factor (RF), and anticyclic citrullinated peptide (CCP) were within normal limits, but his c-reactive protein (CRP) was elevated at 11.6 mg/dL and white blood cell count was elevated at 40.4 × 10^9^/L. He was given an intravenous bolus of dexamethasone 10 mg with marked improvement in joint pain. The patient was referred to a rheumatologist who, due to steroid-mediated symptom remission, elevated CRP, pitting edema of the hands, and improvement with corticosteroids, made the diagnosis of RS3PE.

## Discussion

### Description

RS3PE is a rheumatic syndrome characterized by symmetric synovitis of the hands and ankles with associated pitting edema, negative RF, and elevated acute phase reactants. This diagnosis can present similarly to rheumatoid arthritis, but RS3PE is not associated with subcutaneous nodules or radiographic evidence of erosion.^3^ It is often diagnosed in patients with underlying neoplasia, particularly hematological malignancies, solid tumors, and adenocarcinomas, but it can also occur in patients without underlying malignancy. Almost all patients achieve full symptom remission with glucocorticoids,^4^ and some patients may have complete resolution of symptoms if the underlying malignancy is treated.^5^

### Clinical presentation

RS3PE was first noted by McCarty in 1985, when he described 10 patients who presented with bilateral transient pitting edema and synovitis that were seronegative for rheumatological factors.^6^ Symptom onset was so sudden that 7 out of 10 patients could recall the exact hour at which their symptoms began.^7^ Patients often present with joint pain and bilateral swelling of the hands, which increases suspicion for rheumatoid arthritis; however, most patients are anti-CCP and RF negative. Positive ANA can occur but is nonspecific for RS3PE. Acute phase reactants, such as CRP and the erythrocyte sedimentation rate, are typically elevated.^7,8^

RS3PE mimics several more common rheumatological disorders, but there are key distinguishing features that are more specific to this syndrome. It is important to distinguish RS3PE from polymyalgia rheumatica (PMR); Kimura et al*,* in a retrospective study, found that their cohort of 28 patients who met criteria for RS3PE also met the diagnostic criteria for PMR.^9^ Bilateral pitting edema in the hands and feet is more common in RS3PE than in PMR, therefore, it can be used as a distinguishing diagnostic factor for RS3PE.^10^

X-rays obtained in patients with RS3PE often reveal no joint destruction, which differs from the classic presentation of rheumatoid arthritis.^11^ Intra-articular synovial effusion, mild articular synovitis, and digital flexor tenosynovitis are commonly observed in RS3PE, which can be evaluated using musculoskeletal ultrasonography. Synovitis and tenosynovitis are typically more severe in RS3PE than in elder-onset rheumatoid arthritis, helping to further distinguish RS3PE as a unique syndrome.^12^

RS3PE can occur spontaneously, but an association with underlying malignancies such as CLL, prostatic carcinoma, ovarian carcinoma, and lung malignancies has been reported.^13–15^ It appears that RS3PE can present as a paraneoplastic syndrome, although due to the rare nature of the syndrome, this hypothesis is still under debate.^3,10^ Therefore, a diagnosis of spontaneous RS3PE should be followed by a workup for occult malignancy.

### Pathophysiology

The pathophysiology of RS3PE is still unclear; however, vascular endothelial growth factor (VEGF) is suspected in the pathogenesis. VEGF is an important component in the growth of new blood vessels and in repair, but it has also been associated with multiple disease processes ranging from diabetic retinopathy to cancer cell growth.^16^ Arima et al demonstrated a relationship between the severity of the disease of RS3PE and the levels of VEGF, hypothesizing that both the synovitis and the edema present in RS3PE may be caused by elevated concentrations of VEGF, as increased angiogenesis can result in more inflammation and edema in the joints.^17^

Furthermore, VEGF is inhibited by glucocorticoids, which is one of the mainstays of treatments for RS3PE; thus, the improvement of symptoms from steroid treatment may be a result of VEGF inhibition. Cancers have been known to produce VEGF to promote tumor growth and sustainability of the tumors,^18^ which may be why RS3PE is more commonly seen in patients with malignancies.^19^

Other than the possible role of VEGF in the pathogenesis of RS3PE, data on other potential etiologies is limited. No association has been demonstrated with HLA-B27, a common HLA subtype in other seronegative spondyloarthropaties.^20^ This could suggest an immunological component, but research is lacking.

### Treatment

RS3PE can be managed primarily through glucocorticoids and treatment of the underlying malignancy. In the original description of the syndrome, McCarty described cases in which symptoms resolved after treatment with various medications such as aspirin and hydroxychloroquine, without any clear modality consistently resulting in resolution of symptoms.^9^

Corticosteroids have been shown to be an effective treatment for RS3PE. In McCarty's first description of the disease, multiple patients responded well to low-dose (10 mg daily) glucocorticoids. Patients generally respond well to prednisolone, and its prescription at the beginning of the syndrome has become standard of care.^21^

In addition to glucocorticoids, treatment of the underlying malignancy, if any is present, is likely to improve the syndrome. Case studies involving patients with non-Hodgkin lymphoma and prostatic carcinoma with a coexisting diagnosis of RS3PE demonstrated that treatment of the underlying malignancy often led to an improvement in symptoms of RS3PE.^22,23^ Ferrao demonstrated resolution of symptoms of RS3PE in a patient after removal of a lung mass.^24^ Sakamoto et al published a case study that showed that even without complete remission of the malignancy, chemotherapy led to improvements in symptoms of RS3PE.^25^

## Conclusions

Palliative medicine providers are often consulted for the treatment of cancer-related pain. RS3PE is an uncommon syndrome that palliative medicine providers should be able to recognize and treat. Recognizing it as a distinct cause of bone and joint pain is important, as its symptoms respond to treatment, particularly corticosteroids. Early consideration of the syndrome may result in earlier diagnosis, thus reducing the burden of pain and subsequent opioids in some patients.

## Abbreviations Used

ANA: antinuclear antibody
CCP: cyclic citrullinated peptide
CLL: chronic lymphocytic leukemia
CRP: c-reactive protein
HLA: human leukocytic antigens
PMR: polymyalgia rheumatica
RF: rheumatoid factor
RS3PE: remitting seronegative symmetrical synovitis with pitting edema
VEGF: vascular endothelial growth factor
